# Expanding Aesthetics

**DOI:** 10.3389/fvets.2022.855087

**Published:** 2022-05-04

**Authors:** Fiona French

**Affiliations:** School of Computing and Digital Media, London Metropolitan University, London, United Kingdom

**Keywords:** aesthetics, design, inclusivity, multi-species, perception, Animal-Computer Interaction, animal centered computing, more-than-human design

## Abstract

This paper seeks to expand traditional aesthetic dimensions of design beyond the limits of human capability in order to encompass other species' sensory modalities. To accomplish this, the idea of inclusivity is extended beyond human cultural and personal identities and needs, to embrace multi-species experiences of places, events and interactions in the world. This involves drawing together academic perspectives from ecology, neuroscience, anthropology, philosophy and interaction design, as well as exploring artistic perspectives and demonstrating how these different frames of reference can inspire and complement each other. This begins with a rationale for the existence of non-human aesthetics, followed by an overview of existing research into non-human aesthetic dimensions. Novel aesthetic categories are proposed and the challenge of how to include non-human aesthetic sensibility in design is discussed.

## Introduction

“*What's it like to be a human*

*the bird asked*

*I myself don't know*

*it's being held prisoner by your skin*

*while reaching infinity*

…

*That's funny said the bird*

*and flew effortlessly up into the air”*

- From the poem Funny by Kamienska (1).

Humans have historically claimed five senses - sight, hearing, touch, taste and smell. Each of these has a corresponding aesthetic dimension, in that humans have identified facets of each sensory experience and learned to adjust the aesthetic qualities of designed artifacts so that they give greater pleasure. This has resulted in the development of creative arts such as painting, music, textiles, cooking and perfumery. However, there exist other aesthetic dimensions that are not perceptible to humans because we lack the necessary sensory organs. One example of this is electromagnetism, a phenomenon that humans seem not to be able to detect without using technology. There is increasing evidence that a wide range of animals perceive and utilize electromagnetic fields for global positioning and migration (e.g., birds, sea turtles, wolves, butterflies), and to detect prey and predators and mates (e.g., sharks, skates, rays).

All human senses have a restricted range, because like other species, we evolved to be able to discriminate the sensory information that would maximize our potential for fitness in a specific setting. Too much unnecessary information would be sensory overload for our brains. Even sight, the sense that is most commonly associated by humans with aesthetic quality, has built-in limitations for our species. By contrast, zebra finches possess a tetrachromatic color spectrum, which means they can see extra colors that we can currently only imagine; hedgehogs and eagles can perceive ultra-violet light. These adaptations provide important benefits to their owners, contributing to survival and welfare.

Expanding aesthetics is about exploring the qualities of non-human sensory experiences, despite not necessarily being able to perceive the sensory information in the same way ourselves. As an example, even if it is possible for a human to sense the same stimulus as a bear, our perception of the stimulus and its associated meaning are inevitably different, because of our distinct life experiences. Moreover, we know that many other species have different sensory apparatus to humans and are able to perceive and interact with the world using different modalities. Yet we can use technology to support the capture and analysis of non-human sensory information, and it is possible to take an anthropological approach to gain deeper understanding of the context. This paper also includes some literary references, a reminder of how human imagination has been captivated by the idea of embodying the “other” and on occasion, striven to explore unknown dimensions of being.

Technology has been used extensively to enable humans to interact with the world and perceive phenomena that we are incapable of discerning with our own neurobiological systems. Prosthetics, false teeth, ear trumpets and eye lenses have been around for centuries, supporting proprioception, touch, hearing and vision. The investigation of new dimensions of perception experienced by other species is a more recent area of research that is developing as we make progress in understanding non-human animals. Modern technology includes seismic sensors that can pick up vibrations, algorithms that can translate and applications that can visualize acoustic signals, biochemical tests to identify constituents of a substance, infrared and ultraviolet cameras, pressure sensors and more. Building awareness of non-humans through a range of multidimensional sensory apparatus can help humans to understand the complex needs and pleasures of other species.

Since humans dominate the global ecosystem, it is critical for us to understand the implications of our ubiquitous presence and associated technologies, so that we can design to live equitably with others. Additionally, the knowledge derived through expanding our perceptive and aesthetic capabilities may have relevance beyond the original contexts. The insights accrued may allow us to appreciate what constitutes a joyful moment for a non-human animal and in discovering how to facilitate that, experience the confluence of cognition and emotion that constitutes joy for ourselves. We may become empowered - delighting in gaining new perspectives, with our perceptions enhanced by technology.

Many technology-enabled systems designed for animals have a very specific context – for example, zoo or lab enrichments, systems for livestock, indoor games for domesticated companion animals – which means there are multiple opportunities for designer practitioners to explore the aesthetics of the devices they create. The field of Animal-Computer Interaction (ACI) emphasizes animal-centered design and associated ethics (2) and this paper aims to contribute an aesthetic dimension to the field in the form of a review of current knowledge and some suggestions for exciting future research.

The content is divided into three sections: The first of these offers a rationale for non-human aesthetics, considering how different disciplines have tried to interpret and understand the aesthetic experiences of other animals. The second section offers an overview of existing research into non-human aesthetic dimensions and suggests some novel aesthetic categories relating to patterns of behavior. The third section relates to the challenge of designing for non-human animals and discusses how researchers from a broad range of disciplines might inspire each other through sharing their ideas and perspectives.

## Rationale for Aesthetics

“*Aesthetics could well be an important part of the evolution of life, and consciousness, on Earth, allowing organisms to better interact with the universe surrounding them*.” - Thompson (3).

Do non-human animals experience aesthetic pleasure? And if so, how and why? Possible answers to these questions draw together research from multiple disciplines, including neuroscience, anthropology, philosophy and ecology.

### Philosophical Theory

According to Berleant (4), “*aesthetic appreciation is … a complex multi-sensory perceptual engagement by means of a cultivated sensibility*.” He explains that aesthetic sensibility requires sensory awareness, perceptual discrimination and the ability to discern intensity, evoking cognitive, emotional and potentially physical responses. This perspective is supported by the work of Berlyne (5) who concluded that in conjunction with perception, discrimination and emotional sensitivity, learning and hedonic value are critical psychological processes associated with aesthetic appreciation.

Applying this definition to non-human animals, it follows that aesthetic sensibility relates to an animal's ability to perceive a phenomenon or experience, to be able to discriminate between that and other phenomena of a similar sensorial type, to have the capacity to enact a preference choice, and for this judgement to be motivated by immediate personal experience. This means there is a reward associated with different sensory episodes at the time of experiencing them, and that therefore the potential exists for more or less pleasurable environments and experiences.

### Evolutionary Rationale

At a neurobiological level, Skov (6) states: “*…liking emerges when certain patterns of neural activity in reward structures assign a measure of hedonic value to perceptual representations*.” In other words, we see, hear, smell, taste or feel something that gives us a positive response and we store this information. Skov continues: “…*biological organisms can only come to form preferences for the parts of the surrounding world they can perceive, and these parts themselves are a result of the individual species' evolutionary history*.”

Interest in the biological determinants of aesthetic preference has led to several distinct theories, some of which are grounded in the use of sexual selection as an indicator of aesthetic choice. A brief description of each follows.

Evolutionary Biology (EB) proposes that all traits are directly or indirectly linked to fitness, thus being honest signals to potential partners – this means that the quality of the trait (e.g., size of horns, volume of croak) genuinely corresponds to the reproductive quality of the mate with that characteristic. More often, the chooser in this scenario is the female, because she produces fewer gametes (eggs) than the male (sperm), so logically she should be more selective. Thus, EB states that all aesthetic preferences associated with partners are in fact related to reproductive success. The same reasoning can be applied to all aesthetic preferences about everything, if lifestyle choices are also driven by an evolutionary mechanism aiming to ensure both individual and species welfare and longevity (6).

**Figure 1:**
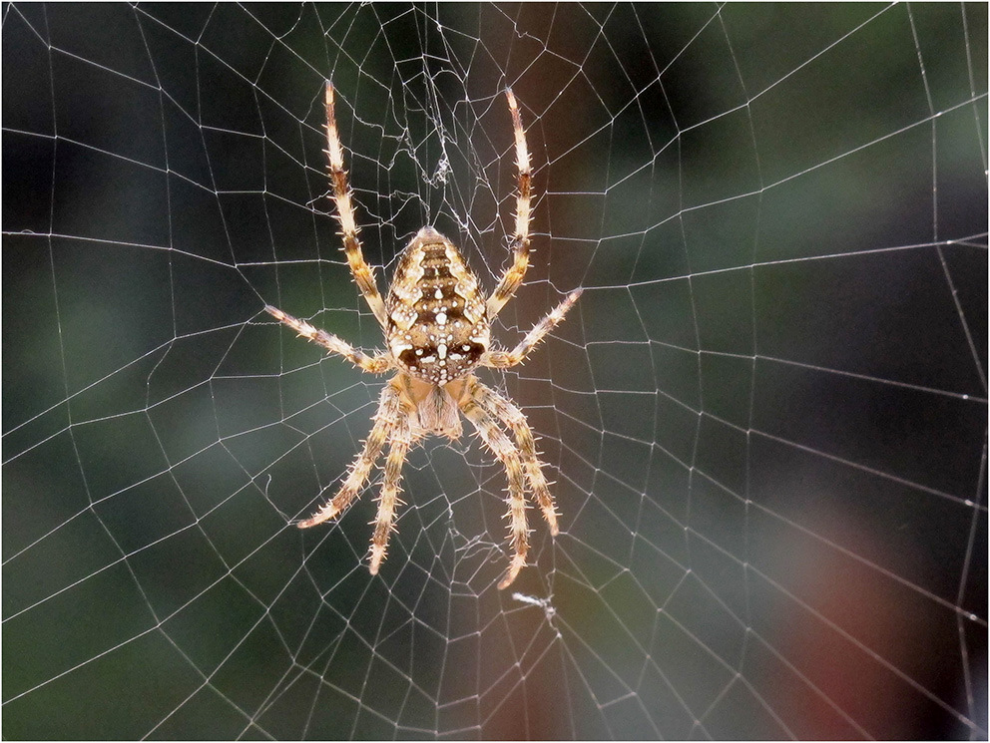
Orb-weaver spider in London garden, UK.

However, it could also be argued that such an explanation undermines the value of cognition and free choice. As Abram [(7), p.50] says: “*Consider a spider… however determinate one's genetic inheritance, it must still… be woven into the present, an activity that necessarily involves both a receptivity to the specific shapes and textures of that present and a spontaneous creativity in adjusting oneself … to those contours*” (See Figure 1: Orb-weaver spider.). Static camouflage, on the other hand, is considered strong evidence of natural selection [since (8)]. Cott (9) identified various forms of predator and prey camouflage that provided concealment in pursuit of food or safety, thereby contributing to fitness.

Supporting EB as the underlying mechanism for all brain function, Pinker famously dismissed music as “*auditory cheesecake”* (10). His super-stimulus hypothesis claimed that music was “*pure pleasure technology”* and therefore “*biologically pointless*”, comparable with pornography. This position has been criticized by other researchers (11–13) who point out that music has adaptive value, since it conveys information between minds and enhances communication and emotional skills.

Another model, based on work by Fisher (14) contests that aesthetic preference is a self-reinforcing phenomenon that occurs as traits become preferred in the population. More individuals with those traits are born, thus increasing the frequency of those traits, usually through selection for the male line. Selection for traits happens over several generations. Prum (15) suggests that in this case, preferred traits are merely “*attractive*” rather than utilitarian; in other words, they are not preferred because they denote fitness in a mate. In human society, this might account for the emergence of trends.

Sensory Bias – (16, 17) suggests that preferences evolved as responses to the environment and were subsequently also used by the neurobiological system that directs sexual choice. Dutton's Savanna Hypothesis (18), based on Orian and Heerwagen, (19) attributes human creative choices in painting and landscaping to evolved preferences for open spaces with visible water, animals and vegetation, because these provide the optimal conditions for human life.

Experiments have demonstrated that sensory preferences occur outside sexual selection (20), and that reactions to specific stimuli can be learned, which means they are based in cognitive processes, not only occurring as naturally selected behaviors. Moreover, preferences depend on context – they are flexible and can be influenced by both internal (e.g., hormonal) and external (e.g., competitive) factors. Different species are therefore likely to have different aesthetic preferences.

Association with intelligence – Watanabe (21) considers three aspects of aesthetic behavior: cognitive, hedonic and creative. *Cognitive* aesthetic behavior includes the ability to discriminate between options, requiring not only the ability to perceive differences, but also to make choices by recognizing and understanding these distinctions. This clearly identifies aesthetic sensibility as a cognitive process while also emphasizing that there is variety in any environment. Thompson points out: “*Intelligence has been strongly selected for throughout the evolution of life on earth, not only for hominids … but for much, if not all, of life... Aesthetics therefore has been strongly selected for throughout its evolution*” (3). In other words, although behavioral responses and variation in genotypes may be driven by evolutionary biology, there is an assumption that individuals retain the capacity to make meaningful decisions and to learn through their experiences.

### Hedonic Ethnology

Watanabe explains *hedonic* aesthetic behavior as deriving from the neurobiological system that rewards pleasurable experiences (22). This has been exploited in training scenarios that use positive reinforcement (from humans) to shape behavior (of humans or non-humans). *Creative* aesthetic behavior might include activities such as decorating, crafting, tool-using, puzzle-solving, playing or performance. All of these creative activities are strongly based in cognition.

Watanabe claims that although human aesthetic creativity (for example, visual or auditory art) can have hedonic value for other animals, such behavior in non-human animals has no reinforcing property for their conspecifics. However, this seems to contradict other perspectives on aesthetic sensibility and the rationale for its evolution. None of the proposed theories discount the possibility that is gratifying to be the *subject* of an aesthetic experience offered by a creator who exhibits inherited preferred traits. Hogh-Olesen (23) states that as aesthetic expression and appreciation is inherent in human nature, it is therefore a primary impulse, requiring no external reward. Nonetheless, although humans spend a great deal of time on aesthetic activities that are apparently unrelated to fitness, these are not useless – the author clarifies that aesthetic skills are valid fitness indicators, providing mating opportunities, higher status and more collaborative offers. From a human perspective, even if we consider an artist to be highly attractive because of their particular skills, there is still pleasure in the moment of experiencing and acknowledging the artistry, beyond any personal connection with the artist. If humans can appreciate a sensory stimulus in and of itself, might not there be an aesthetic reward (experienced as pleasure) for other non-human animals in response to a particular stimulus in their environment, including a stimulus presented by a conspecific?

Balcombe (24) makes a strong case for hedonic ethnology, proposing that aesthetic preference may be distinct from evolutionary drive. He suggests that traditional explanations of behavior rooted in adaptation cause a lack of focus on alternative explanations that are related purely to pleasure. His perspective is that animals are sentient, emotional and aware, capable of experiencing many pleasures beyond those directly associated with fitness (nutrition and sex); these include comfort, visual beauty, play, touch and taste. This perspective is shared by Cabanac, whose research on sensory pleasure included an experiment with an African gray parrot (25). He demonstrated that the parrot was capable of learning words to discriminate between good and bad stimuli and moreover to apply this vocabulary to novel situations relating to types of food. Cabanac suggested that this demonstrated the parrot's aesthetic preferences in its current context, rather than being an evolved behavior.

Affective states (emotions) in non-human animals can be hard to assess but are usually measured in terms of valence (positive to negative experience), arousal (strength of response) and motivational intensity (how much the stimulus provokes a corresponding action) (26). There is wide agreement amongst neuroscientists and biologists that animals ranging from primates to fish experience emotions (27–30), and Balcombe is adamant that humans should not deny animals ‘feelings’ just because we are unable to prove their existence. Moreover, he cautions: “*Because many animals have more acute senses than we do, they may feel certain things more intensely than we do*” (31). As we shall discuss, there are many sensory aspects of life on earth that are imperceptible to humans, with corresponding pleasures for the animals that experience them.

### Anthropological Perspective

Human culture encompasses aesthetic choices, according to Hogh-Olesen (23), who suggests they convey a “*unifying social marker”*. Westphal-Fitch and Tecumseh Fitch (32) also endorse this idea, claiming that humans possess “*culturally coevolved aesthetics”*, which explains the differences across populations. This point is picked up by Thompson (3), and expanded to include all animals, not only humans. He suggests that socially shared aesthetics are responsible for *collective intelligence*, helping to create different cultures within populations of species. Thompson's interest is folklore in anthropology, and the broadening of this field to encompass more-than human communities, part of a recent movement known as the “*animal turn”*. Magliocco (33) expresses this shift in an anthropological context thus:

‘*Is folklore—meaning traditional expressive culture exhibiting variation over time and space—perhaps not a uniquely human phenomenon? Or can it be said to have derived evolutionarily from a set of behaviors common across a number of species? Is aesthetic performance … in fact common to many species, and ultimately rooted in perceptions of the natural world and experiences therein as “pleasant” or “unpleasant?”*’

Thompson makes the point that there is a lack of studies on the aesthetic perspectives of animals, due to a prevailing assumption that the human is the only species to have an aesthetic sensibility. On the other hand, Latini (34) suggests that the challenge for researchers is related to an anthropomorphic tendency to position non-human aesthetics within a human framing, such as “*providing common culture*” or even “*conferring evolutionary advantage*.” However, as suggested, there is currently much interest in exploring the spaces and perspectives of other species (their “umwelten”), as humans become more acutely aware of our global impact during this epoch (often referred to as the “Anthropocene”).

### Ecology and Atmosphere

Lorimer et al. proposed a new concept – animal atmosphere – to describe the geographical space that non-human animals occupy; animal atmospheres are spaces with “affective intensities” of varying types, often derived from scents, patterns and rhythms that humans do not readily perceive or understand (35). The authors suggest that investigating these atmospheres offers researchers a window into “*a rich and underexplored diversity of ways of being in the world*.”

Lorimer et al. explain how sensitive some animals can be to meteorological dynamics, such as perceiving minute changes in pressure, temperature, humidity, light and wind direction. These changes in atmosphere might be critical for motivating particular seasonal behaviors, such as hibernating, mating or migrating. Moreover, the non-human world is full of biochemical signals that we fail to appreciate, spectra that are outside our limits of perception and territories with invisible boundaries. The expansion of aesthetics to incorporate non-human sensory modalities and mindsets is therefore both topical (the animal turn) and highly relevant for Animal-Centered Research and Design.

The following section comprises an overview of current knowledge about animal perceptive abilities, and a speculative discussion relating to behavioral aesthetics and how they might be defined.

## Perception, Aesthetic Sensibility and Behavior

“*It is entirely possible that behind the perception of our senses, worlds are hidden of which we are unaware.”* – Attributed to Albert Einstein.

To illustrate the breadth of perceptual possibilities, the section *Sensory Aesthetics* introduces senses individually, while acknowledging Berleant's observation that: “*…sense perception is never simple sensation or pure perception..*.” (4). Perception as a holistic experience occurring in a particular context is highlighted in the section *Behavioral Aesthetics*, which offers some suggestions for categorizing types of behavior that have intrinsic reward. Readers are invited to consider what aesthetic sensibility might mean in each context.

### Sensory Aesthetics

Humans rely predominantly on vision to evaluate the world (36), but we know this is not the case across species. For some animals, vision is useful, but not a primary sense, while others can perceive more visual spectra than humans, so their sight provides them with dimensions unknown to us.

#### Visual

*In his exploration of octopus evolution, Godfrey-Smith asks: “What could it be like to see with your skin?*” (2016). He explains that the cephalopod nervous system is very different from the vertebrate configuration, in that perception and control is distributed throughout the body, instead of a control center being located in one location – the brain. Twice as many neurons exist in the combined arms of an octopus as in the brain. Although octopuses have excellent vision using their eyes, their limbs are able to dynamically and independently create camouflage. Their skin has millions of photoreceptors that both sense and respond to light, changing skin color and forming patterns in response to the environment. If the skin sense is communicated to the brain, octopus vision extends wherever the arms can reach; if it remains local, then each arm can see for itself.

Mantis shrimps are famous for having 16 color receptors in their eyes, and this allows them to perceive *polarized* light that occurs in different patterns underwater (38). Navigation on land using polarization has been documented in arthropods, but Powell and team developed a video camera that could capture polarized light underwater and render it visible to humans, showing that this information could be used both for geolocation and as a compass. Kelber, in his examination of tetrachromatic color vision in birds (39) states: ‘*Seeing the world “with bird eyes” is very difficult for humans with human* eyes.’ Birds have four color receptors, compared with human three; the extra cone enables them to see *ultra-violet* (UV) light, whose wavelength is outside the human range of perception. Demonstrating the importance to animal welfare of exploring non-human sensory modalities and associated experiences is a recent study by House et al. that showed how rearing chickens in conditions with supplementary UV light lowered their stress and fear levels (40).

#### Infrared

Infrared waves are at the other end of the human visual spectrum, yet it is worth noting that *infrared sensing* is linked to the *somatosensory* system (see below), as it is the detection of temperature, rather than light. Pythons, vipers, boas and vampire bats all possess a heat sensing *pit organ* at the front of their heads, enabling them to generate thermal images (41). Combining thermal and visual images supports the snakes to detect prey extremely accurately. It is thought that vampire bats use this sense to detect specific locations for feeding, where the warm blood is closer to the skin surface of the prey (42).

**Figure 2:**
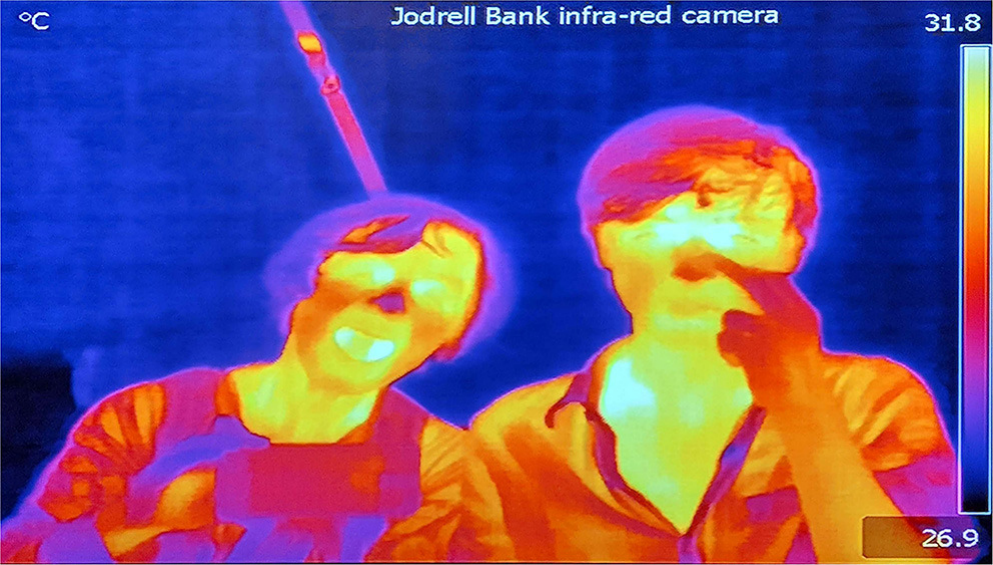
Still from infrared camera at Jodrell Bank, Cheshire, UK.

In captivity, environmental sources of infrared (IR) are usually static, such as heat-lamp-enabled basking spots, whereas in the wild, IR radiation is a more dynamic feature of life. IR cameras can provide humans with a visual representation of this sense, expanding our perceptive repertoire (see Figure 2: Still from infrared camera.).

#### Somatic

Touch is thought to be the first sense to develop (43) and it is fundamental for interacting directly with the world: “*our primary conduit of both pleasure and pain*” (44). As an example of the connection between touch and positive affect in non-human animals, studies have shown that tickling captive rats induces them to chirp as if they were engaged in rough and tumble play with each other, thereby demonstrating their apparent pleasure (45). But tickling is not only a tactile experience; it also relates to the *performance* of an activity and the resulting sensory feedback for both parties. Godfrey-Smith explains: “*In everyday experience there are two causal arcs. There is a sensory-motor arc, linking our senses to our actions, and a motor-sensory arc as well…. The effect of action on what we sense next is surely important*” (37). Abram similarly emphasizes reciprocity through physical performance: “…*perception, experientially considered, is an ongoing dynamic…*” (7), p.81. Proprioception (kinaesthesia) is the awareness of bodily movement, but performance is the enabler of other sensory experiences, and has its own aesthetic dimension (46, 47).

In humans and other animals, somatic sensation arises from the body surface or internal organs and endows us with the sense of touch, proprioception, pain (nociception) and temperature (48). Linden (43) explains that in humans, there are two distinct systems for touch: (i) a discriminative sensory pathway that provides information about vibration, pressure, location and texture; (ii) an emotional pathway that processes pleasure, pain and social information related to the sensation experienced. It seems likely that the confluence of these signals is necessary for aesthetic appreciation, and moreover, that similar systems exist in other animals who share our evolutionary neurobiological roots. Research by Gibbon et al. (49) indicates that bees can modulate their nociceptive responses to prioritize feeding, which suggests that these are insects capable of perceiving pain. If so, does this point to a capacity for also experiencing pleasure?

**Figure 3:**
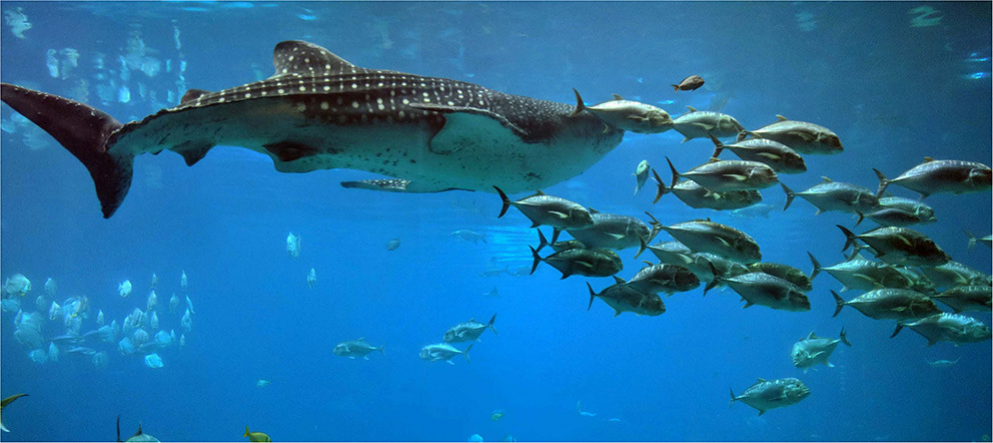
Whaleshark and shoal of golden trevally in Georgia Aquarium, USA.

Some species are acutely mechanosensitive, with specialized organs for tactile perception. Fish have a lateral line, which is a series of pores along the length of the body that can sense pressure changes, and by association, movement and vibration. The lateral line detects lower frequencies (less than 100 Hz) than the auditory system. It is thought to play an important factor in schooling, by providing information about neighboring fish and facilitating synchronized movement (see Figure 3: Whaleshark and shoal of golden trevally). In this way, the lateral line increases the ability to detect prey and also supports a mechanism for prey avoidance, since by swimming together, shoals of small fish generate complex water movement that may confuse their predators (50).

In the case of sharks, their body movement gives them spatial awareness and navigational ability; they create waves that bounce back from obstacles and are detected by the lateral line, providing them with a pressure map of the surrounding environment (51). Crocodilians also possess a pressure sensor – a series of integumentary sense organs (ISOs) in their skin, which are highly concentrated around the jaw area. The ISOs are specialized for detecting touch, particularly in the context of water vibrations that predict and identify prey (52). These senses seem likely to contribute to the complexity and excitement associated with hunting.

On land, the star-nosed mole is a “*somatosensory specialist”*, according to Catania (53). The mole's 22-fingered snout-star acts like a *tactile eye* and has a correspondingly large area of somatosensory cortex devoted to its representation. Snakes, meanwhile, can detect vibrations in the air and through the ground via their body surface, known as *somatic hearing* (54). In their work on tactile intelligence, Liu et al. point to the need for further research in this area: “*The inherent characteristics of tactile signals have not yet been fully explored*” (55).

#### Infrasonic

Large mammals, including elephants, giraffes, rhinos and whales (56–59), communicate using low frequencies that are outside normal human hearing range, below 20 Hz. This is known as infrasound and the sound waves generated can propagate through air, water and earth with less attenuation than higher frequency waves, thus are used for communication across long distances. We note that there is a strong link between tactile and auditory modalities, since both types of perception involve sensors that are triggered by vibrations.

“*…you can touch the speaker cone and you will literally feel the infrasound, far below your hearing range. It's really surprising, like having a new sense*.” - From http://techlib.com/area_50/infrasound.htm by Charles Wenzel.

**Figure 4:**
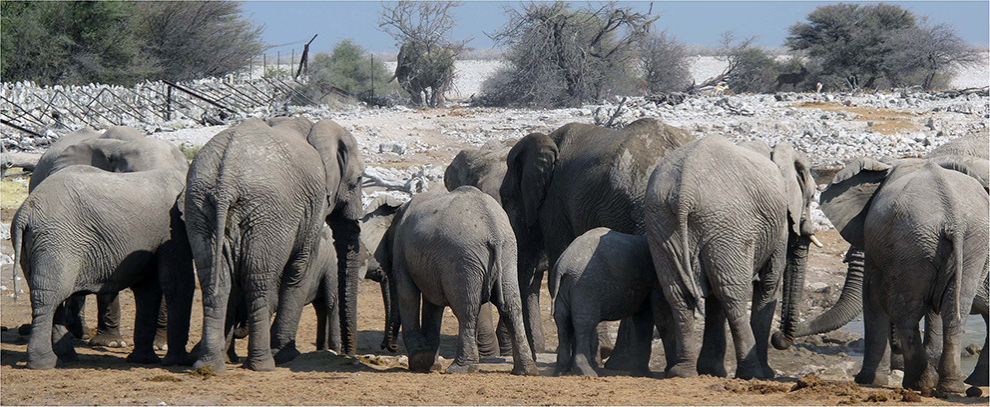
African elephants at waterhole in Etosha National Park, Namibia.

Elephants can detect infrasound through auditory perception via inner ears and also through somato-sensory perception of vibrations via mechanoreceptors in their feet. This may enable them to triangulate seismic information and thereby determine the distance of the sound origin (60). Low frequency vibration traveling along the ground maintains its integrity well, and O'Connell-Rodwell et al. believe that elephants can distinguish the rumbles made by their conspecifics from other background noise (61). Within their herd, elephants exchange these rumbles regularly, known as antiphonal calling (62); giraffes perform a similar aural activity at night, when they hum together (57). The exchanges seem to have social value, promoting cohesion and establishing personal identification within the herd (see Figure 4: African elephants at waterhole).

#### Ultrasonic

At the other end of the acoustic scale, ultrasound is beyond the upper limits of the human hearing range but perceived and generated by many other species. Detecting technology was first used by Griffin in 1944 to monitor bats' echolocation signals in the range 12–160 kHz, based on previous work by Pierce [in Brudzinsky, (63)]. The ultrasonic detection of rat chirps (50 kHz) mentioned earlier led to the discovery of their enjoyment of tickles; this exemplifies one way that improving human understanding of other species' aesthetic responses can potentially lead to better welfare.

#### Musical

“*And the songbirds are singing,*

*Like they know the score”*

- Lyrics from Songbird by Christine McVie, Fleetwood Mac.

It is well established that animals communicate with conspecifics and also glean information by attending to acoustic cues in the environment, but do they produce or listen to sounds purely for pleasure? As Honing asks, in his quest to establish musicality in animals: “*Does a bird hear bird sounds as music?*” (64). According to musician Hollis Taylor, who has recorded songbirds for many years, the answer is yes (65); Gupfinger and Kaltenbrunner, who designed acoustic enrichment toys for gray parrots, describe their users as expert musicians (66); Hoeschele et al. point to entrainment (the ability to synchronize movement to a rhythm) and vocal learning as evidence of musicality in some birds (67). An example is Snowy, a cockatoo who performed spontaneous and diverse movements to music, demonstrating complex planning associated with dancing more than bobbing to a beat (68). Research with cockatiels (69–71) has shown that they improvise with musical toys, are capable of learning melodies and rhythms, and can spontaneously adjust their output so as to sing in unison with humans and other birds.

Recent research into origins of human music shows that our appreciation of tones and harmonies is an evolutionary development linked to our ability to perceive and understand human vocal sounds (72). This makes sense, as biologically having an attraction to sounds emitted by humans and also developing keen discernment of the underlying frequencies and harmonics enables us both to distinguish people by voice, and to interpret emotions, facilitating sophisticated communication. Dissanayake describes music as a “*behavioral and motivational capacity*”, linking our musical development to affiliation through an evolved propensity to respond to other humans' rhythms and sounds (73). If this explains why humans appreciate human-made music, it also likely explains why human-made music holds little interest for the majority of other animals. There are some exceptions; Watanabe and Nemoto tested musical preference in Java sparrows (74), finding that they preferred Bach to Schoenberg and Vivaldi to Carter. While this does suggest that the sparrows have musical preference, the authors' conclusion that the birds prefer “*classical music*” is unfounded. Some musicians have attempted to compose *species-specific music*, notably for racehorses (75) and cats (76), claiming that it has a positive, calming effect. Truax and Vonk (77) emphasize the importance of assessing auditory preferences before introducing acoustic stimulation, which acknowledges the pervasive quality of sound as well as the potential for individuality. Honing has suggested that our attention as researchers should move away from music (interpreted by humans as melody and rhythm) and toward musicality – the ability to perceive relative pitch and regularity in beats (64).

#### Rhythmical

“*And rhythm is all deliciousness; And joy is in the throbbing tide … and music is the exquisite knocking of the blood.”*

– From the poem The Fish by Rupert Brooke (78).

Thompson suggests that through aesthetics, an organism can “*involve itself in the mathematical regularities of the universe”*, citing seasonal changes, cycles of day and night, soundwaves and rhythmical movement as some facets of life on earth that can be represented numerically with accuracy (3). Brando and Buchanan-Smith advocate for animal welfare that takes the patterns of natural life cycles into account (79), while Coe and Hoy (80) argue that abandoning schedules can offer captive animals a kind of relative freedom, allowing for control and self-sufficiency within a population. Captive environments need to be carefully managed, but for a wild animal, there is no such thing as a regular feeding time, for example, although natural activities, such as hunting and foraging, have their own rhythms and chronologies. A tightly managed lifestyle can lead to over-reliance and boredom, potentially contributing to stereotypical behavior.

Temporality therefore has many facets. As well as relating to life cycles and rhythm, we notice that there is a connection between time and olfaction for animals with a good sense of smell.

#### Olfactory

Thwaites has claimed that animals have no sense of history and future (81). He states that only humans conceptualize the world using narratives and that this is what makes us unique. However, because humans primarily rely on vision, we perceive what is around us *at the moment*. For animals who rely on their sense of smell, such as elephants, dogs and bears, there is a connection between time and olfaction. Although our memories and imagination let us traverse time fluidly backwards and forwards, our olfactory limitations require us to live in the present with respect to our immediate perceptions. Dogs, on the other hand, inhabit a world of layered timelines, whereby their noses provide them with complex information about the history of the environment. Scents dissipate over time, so the intensity of a smell is a clue to its age. Olfaction thus provides an example of a sense that informs different species in different ways.

In land vertebrates, the olfactory receptor cells are located in the nasal cavity. Different species possess differing numbers of genes responsible for their activation. Sea dwelling mammals use a dorsal blow hole for breathing into lungs so they can open their mouths underwater. Bovet notes that olfactory activation genes are completely absent in dolphins, although still present in whales (82). Fresh water hunters, such as shrews and star-nosed moles, have adopted a different approach – they blow bubbles beneath the surface and suck them back in again quickly to capture the scent (83). Fish, meanwhile, breath through their gills, but also have noses and are extremely sensitive to waterborne chemicals; any toxic contamination has a negative impact on fish olfaction and subsequent behavioral responses (84).

Majid (85) suggests that a deficiency in vocabulary (in English language) may account for the common belief that human sense of smell is poor. Since humans share ideas and express thoughts through language, lack of words may affect our critical thinking around the topic of olfaction. Moreover, he claims that humans have higher odor sensitivity – meaning lower detection threshold – than animals such as dogs and pigs. In fact, in many cultures around the globe, languages are enriched with olfactory words, notably in hunter-gatherer communities where there is more contact with the natural environment and people possess more ethnobiological knowledge than typical city-dwellers. An example from contemporary western culture is Suskind's fictional account of a man gifted with exceptional olfactory skills, “*Perfume: The Story of a Murderer”*, which was originally authored in French. The English translation is renowned for its literary evocation of odor, albeit using comparisons with known substances rather than a specific olfactory lexicon.

“*This scent had a freshness, but not the freshness of limes or pomegranates, not the freshness of myrrh or cinnamon bark or curly mint or birch or camphor or pine needles, nor that of a May rain or a frosty wind or of well water … and at the same time it had warmth, but not as bergamot, cypress, or musk has, or jasmine or daffodils, not as rosewood has or iris.”*- From Perfume by Patrick Suskind (86) (reprint).

This all points to the need for flexibility in our approach to olfaction and suggests that researchers, writers and designers should not neglect olfactory attributes of systems, even if they seem difficult to describe and quantify.

There are two different pathways to the back of the nose, where the olfactory receptors are located – orthonasal, meaning via the nostrils, and retronasal, via the back of the throat. Dogs, for example, who detect scents orthonasally, can gain information and pleasure from sniffing the environment. Humans have a more developed retronasal pathway, which means we can enjoy the smell of food even more when we put it in our mouths.

#### Gustatory

For those animals that possess a sense of smell, olfaction is strongly associated with food. Olfactory stimuli are integrated with gustatory stimuli when we eat, so that the overall impression of taste is stronger. Humans can detect five tastes with their taste buds – sweet, sour, salty, bitter and umami. While we each have around 10,000 taste buds on our tongues, cows have around 20,000. The increased amount is thought to enable cattle to identify suitable food as they graze, spitting out toxins before ingesting; ruminants show the strongest preference for umami, followed by sweet taste (88). Catfish have 100,000 taste receptors, with concentrations around their barbels. This is an excellent adaptation for finding nutrition in dark, murky water (89). Probably the most advanced sense of taste belongs to the octopus, whose 8 arms each have around 280 suckers, every one with a sense of touch and of taste. There are approximately 10,000 taste receptors on each sucker (37).

According to Balcombe in (24), “…*the experience of food pleasure in animals is almost wholly unexamined*” (24). However, 12 years on, there have been studies with pigs (90), cows (88), fish (91), horses (92), cats (93); dogs (94) and tortoises (95). This demonstrates the contemporary interest in animal wellbeing, which highlights positive affective experiences as fundamental aspects of health and fitness (96, 97).

#### Multimodal

It is important to remember that all our senses are involved with the appreciation of food. Taste is somewhat limited in that it seems to consist of only five detectable tastes in various combinations. *Flavor*, on the other hand, is multimodal, including smell, sight, sound and touch (98). Sight relates to food presentation (e.g., color and shape); sound relates to qualities experienced during eating (e.g., crunchiness); touch relates to mouthfeel [e.g., texture, viscosity, temperature, chewiness, astringency and irritation – (99)].

“*If a French crepe were to marry an English crumpet, the couple would probably become the proud parents of a Sri Lankan hopper. The hopper has the softness, delicacy, and pliability of the crepe teamed with the airy, hole-filled, puffy, and browned-on-the-outside quality of the crumpet*.” – From Eastern Vegetarian Cooking by Madhur Jaffrey, (100).

Perception usually involves multiple modalities perceived simultaneously, providing a holistic experience of an event (101). We are able to integrate the unimodal stimuli associated with a particular event, despite there being other stimuli present. Experiments have demonstrated a superadditive effect, such that the sum of the whole integrated sensorial experience is greater than the sum of the individual parts. In humans, the neurons that process an individual sense send their information to a convergence zone, where all matching perceptions are processed together. Neural convergence happens when there is more than one input neuron sending information to a single neuron; it has been established that the receptive fields around the input neurons (that each only respond to one kind of stimulus) must overlap in physical space in order for the super additive response to be invoked. There are many areas of the brain where this multisensory processing can take place, suggesting that our experience of the world is “fundamentally multimodal”. Studies undertaken thus far with non-human animals indicate that their experience is similarly holistic – examples being cats (102), rodents (103), macaques (104) and flies (105).

The different sensory modalities we perceive can affect each other (101). Multimodal phenomena include perceptions combining to *enhance* a signal, such as the smell and taste combination previously discussed. Another example is found in human speech, where acoustic and visual stimuli support each other from the perspective of a perceiver who lip-reads to capture conversation in a noisy room.

#### Electro-Magnetic

Electro-magnetic field sensitivity is another phenomenon that most humans do not perceive. There is increasing evidence that a wide range of animals can detect and utilize electro-magnetic fields, to determine location and direction, and to detect prey and predators and mates. Animals sensitive to these signals can discern tiny changes in intensity or direction (106).

Clarke et al. showed that bees produce an electrical signal that facilitates pollination (107). The positively charged bee attracts more pollen dust and becomes a better transporter of pollen from flower to flower, but the charge is also detected by the plant. In response, the plant produces more volatile organic compounds (VOCs, otherwise known as scents) that attract more bees. In addition, flowers exhibit electric fields that endow different parts of their anatomy with different charges; petal edges and stigma have a high charge, revealing the overall structure of the flower to an approaching insect.

**Figure 5:**
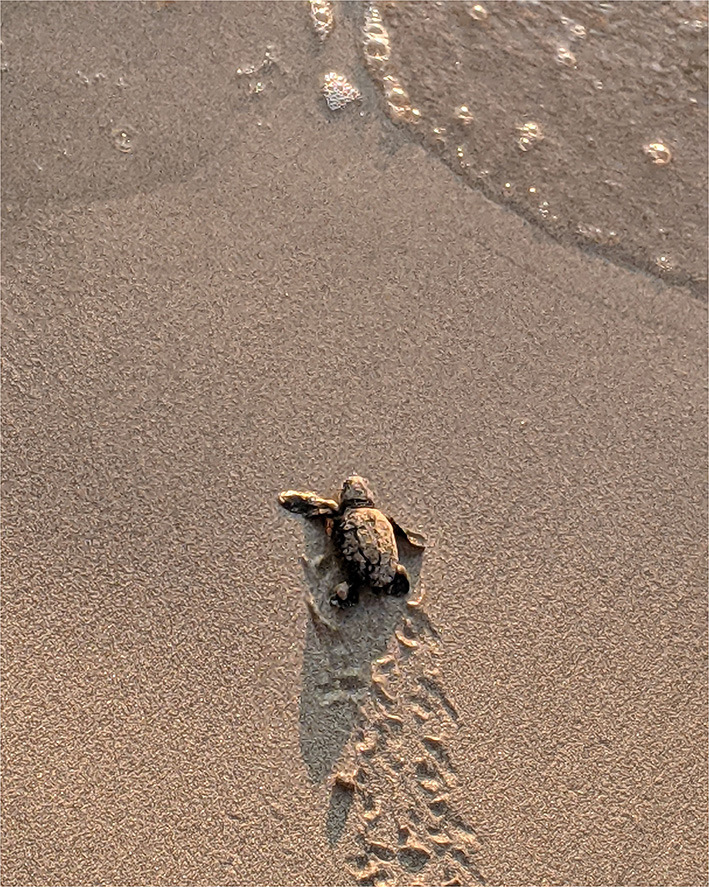
Loggerhead hatchling heading for Mediterranean, Kephalonia, Greece.

Sharks have electro-sensory receptors in organs around their head and mouth. They are highly sensitive to electric fields and can detect muscle contractions in potential prey, as well as the Earth's geomagnetic field (https://www.sharktrust.org/shark-senses). Sea turtles (see Figure 5: Loggerhead hatchling) also use magnetic field information for natal homing (108). According to Clarke et al. (107), there are so many electric fields in the environment that “*signals in this modality could potentially be used by a broad range of species in an array of contexts*.” The corollary is that any interference (such as electromagnetic pollution caused by wireless communication) can have a profound effect.

The range of sensory modalities covered here should give the reader an idea of the extent of aesthetic possibility that might exist for other species. As discussed earlier, action is inherent in all interactions with the environment that result in perceptions, since perception itself is dynamic, evoking a response from the perceiver to a stimulus. At some stage, there is a transition; movement changes from being a single instinctive action to becoming part of an established and recognized behavior.

### Behavioral Aesthetics

Movement facilitates and enhances perception using other senses, and also offers embodied pleasure. Working with elephants led French et al. (47) to the idea of *performative aesthetics*, through observing the animals' preference for interacting with moveable features in their environment. This idea is now expanded to include a wider range of behaviors and phenomena that arguably have their own distinctive aesthetic dimensions for the animals involved.

An important aspect of behavioral aesthetics is that there is a narrative element to the activity that may be missing from a momentary sensory perception. Huron's ITPRA Theory (109) relates to the emotional responses evoked by events that unfold over time. It is a psychological theory of expectation that proposes five contributing systems: (i) the *imagination* required to predict the future in order to make choices in the present; (ii) the *tension* experienced preceding an anticipated event; (iii) an immediate response to the accuracy of the *prediction*; (iv) the feelings associated with the *reaction* to the event; (v) and the final *appraisal* when the outcome is assessed. The emotions experienced by animals during the performance of the following behaviors may fit well with this theory.

#### Aero-and-Hydro-Dynamic

“*Feet, for a flying bird, are an acknowledgment of inadequacy*.”

- From The Screaming Sky by Charles Foster (110).

**Figure 6:**
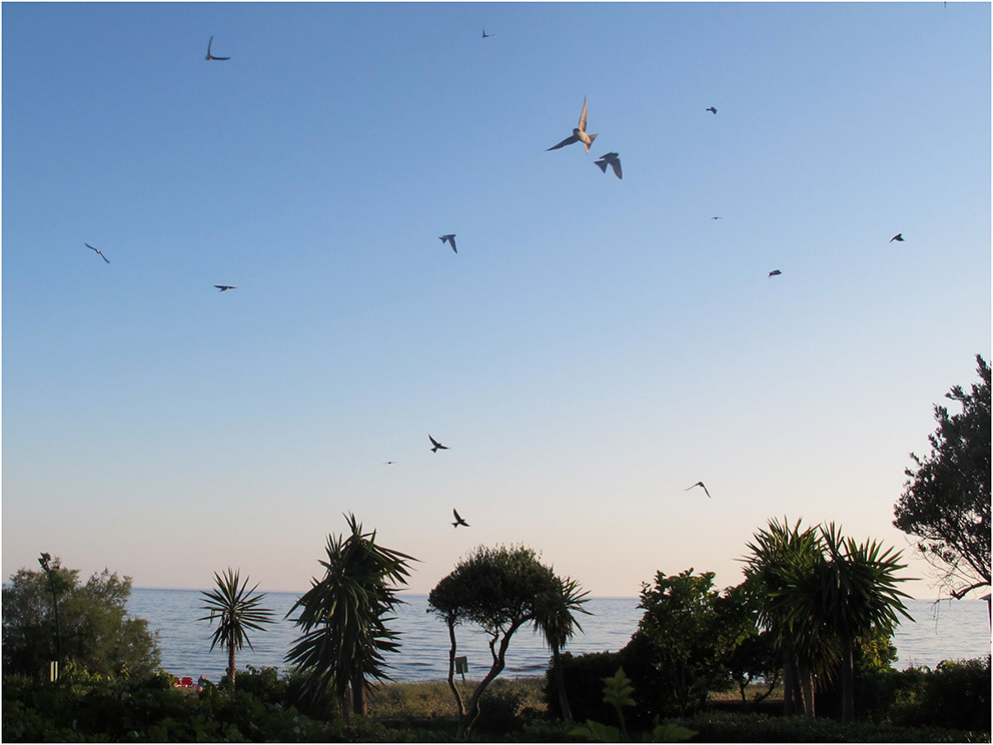
Swifts over Corfu, Greece.

An aesthetic experience that may be hard for humans to appreciate is the combined control and freedom of movement associated with traversing a medium that offers an upward force to counteract gravity. (See Figure 6: Swifts over Corfu.) The ability of an animal to flow in this manner through air or water has been called *buoyancy* for those that are expert fliers, swimmers and swingers. These animals have evolved to be able to move, detached from the ground, with minimum effort and maximum effect. To human observers, such activities appear to elicit joy, to the extent that we have historically tried to emulate the effects, and if not possible, gained pleasure from watching the aerobatics. Abram comments: “*I feel the stretch and flex of its wings with my own muscles, and its sudden swoop toward the nearby trees is a visceral as well as a visual experience for me”* (7), p.61. For animals who normally swoop, glide and go with the flow of their environment, it is often the case that captive conditions are too restrictive to allow for these kinds of movement; for example, aquaculture, which is globally the fastest growing food sector (https://www.fishwelfareinitiative.org/), faces criticism for subjecting fish to overcrowded conditions with associated health problems (111).

Hodgetts and Lorimer point out that mobility is shaped by each species' physical and cognitive characteristics, as well as their habitat (112). It may also be a collective experience, influenced by social factors.

#### Collective

“*An evening murmuration is more than just the dance of starlings; it is a glimpse into one of the fundamental motions of life*.” – King and Sumpter (113).

**Figure 7:**
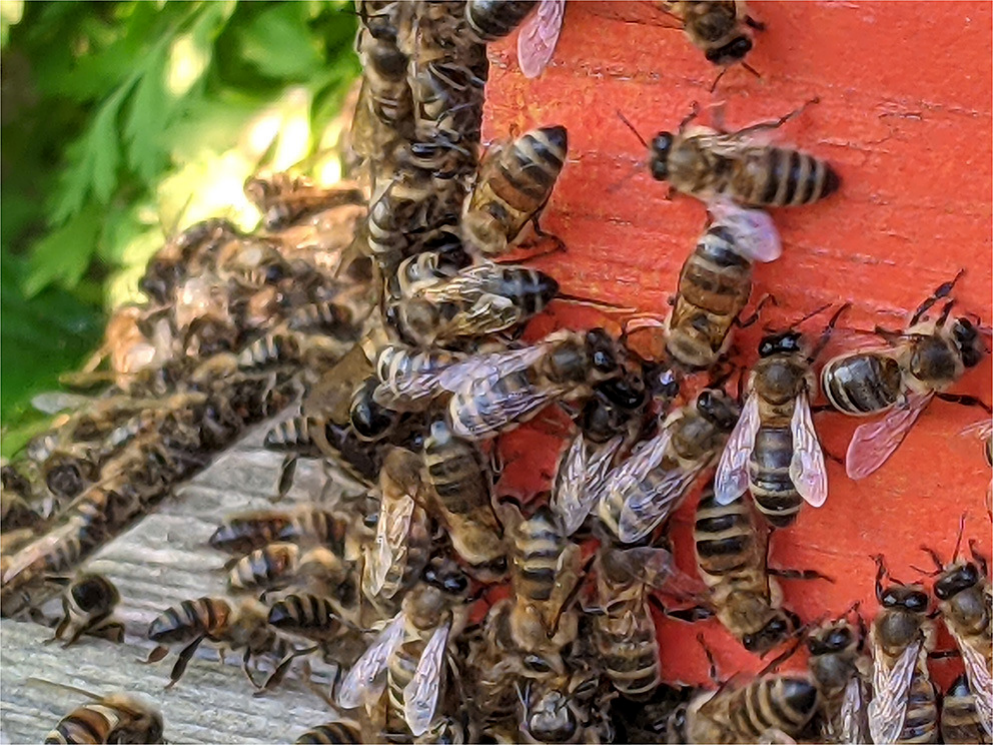
Honeybees in Kent, UK.

Associated with flight but encompassing a different aesthetic, the phenomenon of swarming is exemplified by the murmuration of starlings and the energy of bees (see Figure 7: Honeybees). This kind of performance is a collective behavior that occurs within a system composed of multiple entities that act independently while still maintaining a flow of information between participants (114). The resulting complexity of the system is an emergent property that cannot be predicted by just studying the components as individuals. In Vester Flights (115), Helen Macdonald tells readers: “*Turns can propagate through a cloud of birds at speeds approaching 90 miles per hour, making murmurations look from a distance like a single pulsing, living organism*.”

Emergence seems to benefit both the individuals and the species. In human society, there are health benefits (practical and psychological) associated with being part of, and contributing toward, a bigger system. For a species that lives as part of a colony, collective behavior can give rise to extraordinarily complex results; termite mounds are a case in point, unique structures built cooperatively without any obvious blueprint. There seems to be communal intelligence amongst the participants of a collective behavior, which Sumpter attributes to a set of governing principles, including individual variation, positive and negative feedback, and catalysts (individual influencers) within the group (114). As Werber's fictional etymologist comments, in Empire of the Ants: “*It must be an incredible feeling to live the experiences of others and make them feel everything one feels oneself* ” (116).

#### Playful

“*Play is a process, not a static state of affairs.”*- From The Aesthetic of Play by Brian Upton (117).

Playing is also an activity, performed in a group or by an individual, that arguably has its own distinctive aesthetic, incorporating all the senses of the engaged animal. Upton values choice and agency as the primary aesthetics for play, a position that is challenged by Sharp et al. (118), who point out that making decisions that lead toward the accomplishment of defined goals is not necessarily rewarding. As Greaves comments: “*Very open-ended expressive-responsive movements of (animal) play do not primarily manifest as functionality. Yet they are prime occasions for aesthetic appreciation, both on our part and often on the part of animals themselves*” (119).

**Figure 8:**
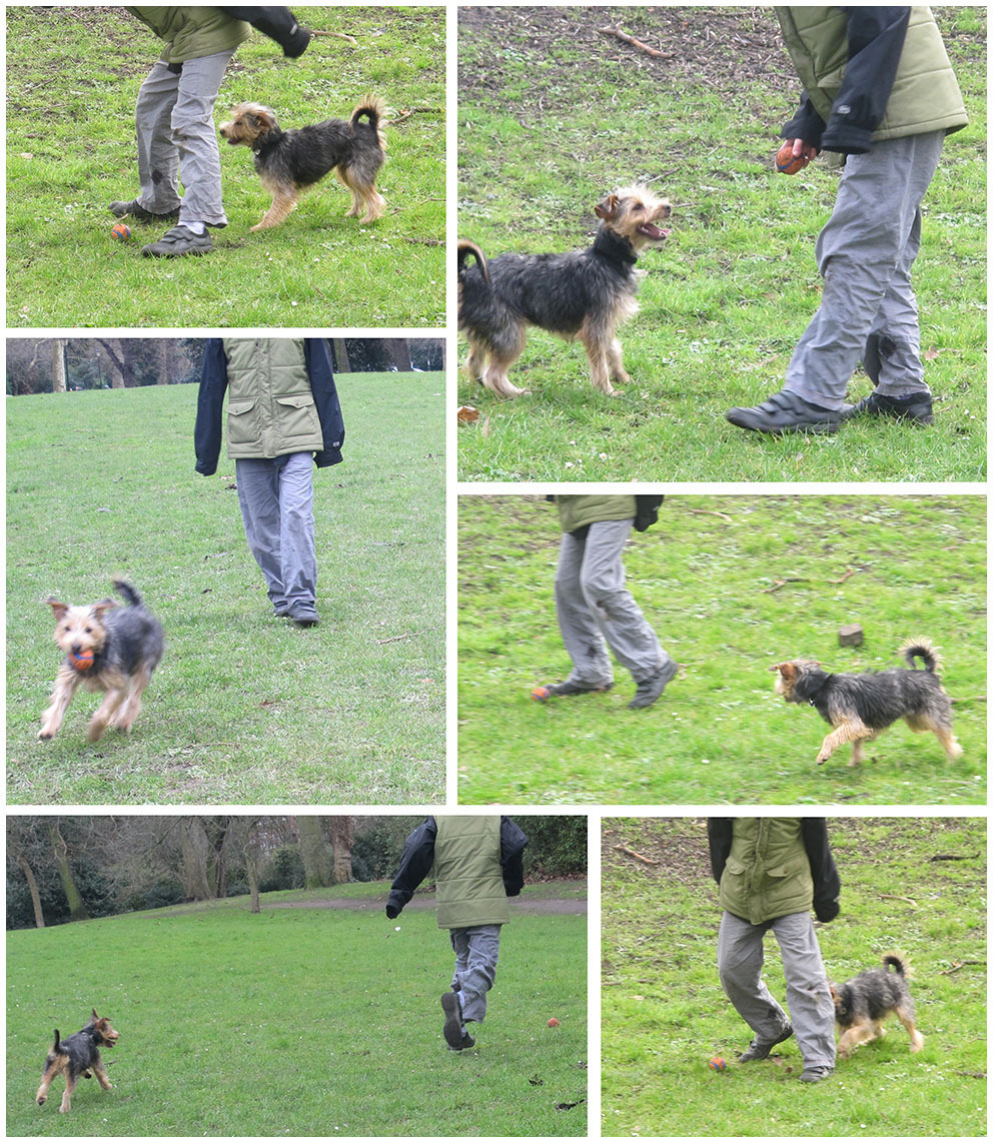
Terrier playing ball with human, London, UK.

Non-human animal play may be easy to recognize but has proved difficult to define. However, as is the case with humans, there exist implicit behavioral rules that participants understand and communicate to each other; this is clearly seen within the frame of human-dog interspecies play (see Figure 8: Terrier and ball) and can be observed in play between other animals (120, 121). One of the “aesthetic ideals” of human gameplay explored by Lundgren et al. (122) is the idea of play emergence, which explains how complexity and interest often arise in social play, despite the rules of engagement being simple. Responses to the constantly changing playscape require players to be alert and cognitively flexible.

Animal play has been categorized as “object”, “social” and “locomotor” (123). Locomotor and object play seem to map very clearly to pleasurable kinaesthetic and tactile experiences, exemplified by the exuberance of spring lambs and the mud-rolling of elephants. While there are welfare-related explanations for such activities – promotion of muscles development and skincare regimes – it seems likely that the play obtains satisfaction for the animal in and of itself. In other words, it is an autotelic activity, self-rewarding on multiple levels.

*Flow* has long been associated with the particular mindset that games can engender in players – characterized as an optimal experience that exhibits high levels of focus and enjoyment (124). For game designers, inducing a state of flow has often been seen as the ultimate challenge, summarized by Salen and Zimmerman as a call to “*design meaningful play*” (125). Although this sounds like a positive objective, there may be ethical issues associated with manipulating players, both human and non-human, so that they invest a large proportion of their time on a designed activity.

Recently, another optimal psychological state has been defined – *clutch*. This is also associated with heightened concentration and performance, most commonly in respect to athletes. In comparing the two states amongst people exercising, Swann et al. (126) comment: “*Flow occurred in contexts involving exploration, novelty/variation, and flexible outcomes, while the experience was described as enjoyable at the time and involved lower perceived effort. Clutch states occurred in contexts involving achievement and pressure. Exercisers perceived clutch states to be enjoyable afterwards but not at the time, and to involve intense effort.”* We argue that clutch pertains fully to the experience of hunting, included here as its own aesthetic category since it is such a fundamental aspect of predators' lives.

#### Predatory

**Figure 9:**
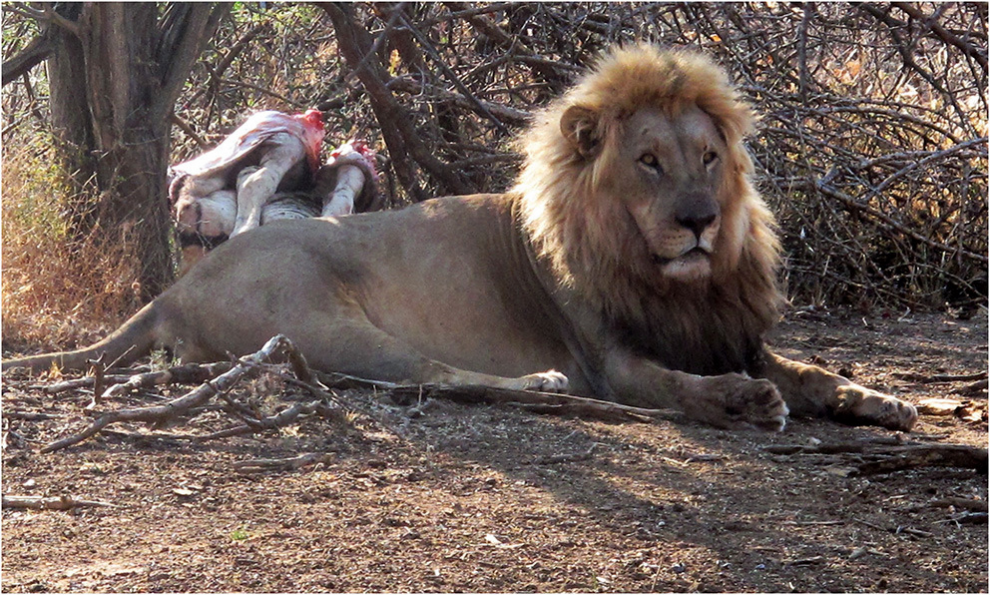
Lion with zebra carcass, Waterberg Plateau Park, Namibia.

Hunting is an activity that completely absorbs the brain and body so that the hunter is in a state of flow or clutch, with heightened perceptions and reflexes. For a predatory animal, hunting facilitates the multiple dimensions of pleasure associated with nutrition, including anticipation, identification and retrieval, ingestion and flavor, and the satisfaction experienced after consuming a meal (see Figure 9: Lion with zebra carcass). In the case of felids, for example, hunting comprises locating food, through traveling and detecting prey; capture, which might entail stalking, coursing, ambushing or scavenging; killing through disabling and dispatching; eating and subsequently processing (127).

Hunting can also be an important aspect of social life and welfare. For social species, pack hunting requires sophisticated communication and coordination amongst the group members, resolving itself in the sharing of the kill. As an example, neighboring groups of bonobos with overlapping territories in the Congo Basin have developed distinct hunting cultures, focusing on different prey to avoid competition between the groups for food (128). On a hunting expedition with dogs (searching for wild pigs), Keil describes how the animals' perceptions enhanced those of the human companions: “*A hunter immerses themselves in the multi-sensual immediacy of their world, attentive to how hunter and hunted affect each other… Chemical, electromagnetic, acoustic, meteorological and other material aspects imperceptible in an environment perceived by naked human senses, can be sensed by nonhumans*” (129).

It can thus be difficult to provide opportunities for captive predators to express their full repertoire of hunting behaviors, since the provision of live prey is not considered ethical in many places, space is restricted, and animals' autonomy is also limited. While there are undoubtedly many excellent examples of captive carnivore enrichment in zoos and wildlife parks around the world, this nevertheless remains a challenge.

### Architectural

“*A bird and its nest belong together so absolutely in our minds that the idea has gone beyond biology and become a motif in the work of poets.”*- Jurgen Tautz in Animal Architecture by Ingo (130).

There are many examples of animals that construct objects from found material or personal secretions, usually as shelters or traps. Notable structures are beaver lodges, which involve serious hydro-engineering and landscape architecture. The attention to detail accorded by beavers to designing, building and maintaining their lodges has been well documented, as well as the associated positive ecological effects on habitat and biodiversity (131, 132). As Laidre comments: “…a*rchitecture changes the world*…” (133).

Birds' nests may be crafted by weaving, excavating and sculpting. The material varies with the environment and size of inhabitants, and the form derives from the function. While it is possible to acknowledge the artistry that goes into building these constructions, we cannot know if the builder derives a sense of satisfaction from a well-made nest. However, in many cases, nest-building is an act of courtship, and for bowerbirds, the selection process has favored visual complexity (134). The male places decorative objects around the bower, selecting specific colors, sizes and positions so as to create an impressive display. Endler comments: “*Great Bowerbirds are artists, judge art, and therefore have an aesthetic sense*” (134). An equivalent behavior has been documented in male puffer fish, who spend many hours constructing geometric circles in the sand to attract females (135).

Finally, this paper presents a small selection of creative methods that have been used by humans to explore the aesthetic dimensions experienced by other species. Not all approaches are directed toward an interaction design challenge, but they all involve imagination, innovation and background research. They may therefore be inspirational for future development in this field.

## Interaction Design: Exploring Aesthetics

Understanding the users of a new system is a priority for interaction designers, but how can they gain empathy and insight into non-human experiences without relevant sensory modalities and world view? Useful methods deployed at the start of any project involving non-human animals include *ethnographic studies*, background *literature reviews*, and *collaborations* with species specialists and animal welfare experts. But is it possible to ask non-human animals for their opinions?

Although some animals can be trained to interpret some human speech, humans have made little progress in interpreting the vocalizations made by non-humans. There is also an assumption that the cognitive processes of non-human animals are less abstract than human thought and therefore less able to be expressed in a human-type language that is highly organized, symbolic and referential. In consequence, interspecies communication is often based on the communication of non-linguistic signals. It may be that different species can understand each other best through mutual observation of expressive behavior. Aspling et al. (136) refer to “*kinaesthetic empathy”* whereby meaning is constructed through bodily experience, and interaction between participants consists of physical movements (137).

However, in the case where human and non-human are not able to interact physically with each other, the provision of *choice* and enablement of *volition* are both crucial for allowing other species to express preferences. Having greater control over their environment is widely recognized as being beneficial for captive animals (2, 138–140), and it is therefore possible to apply this principle to the evaluation of design aesthetics. Ideally, two parallel events should be occurring – the choices made by designers that influence the experience offered to the animals, and the choices made by animal test subjects when they are offered a way to express their preferences. This suggests an iterative mode of development that values incomplete solutions as sources of inspiration and knowledge. In regard to preference testing, paired-choice testing has been criticized because participants may be forced into selecting the lesser of two unpleasant options, rather than necessarily selecting for a hedonistic experience (141). It is therefore recommended to create a range of options, including the option to avoid an experience altogether, as demonstrated in ACI projects with elephants (47, 142) and sakis (143).

To complement an experimental scientific approach, ACI designers have traditionally explored working methods that facilitate empathy and collaborative practice, including all the stakeholders associated with a new system. Another important feature of ACI is that technology has enabled the development of novel tools for designers, such as automated systems and machine learning (ML) algorithms for recognition of behavioral patterns. For example, ML has been used to support the investigation of musicality in birds, through synthesis of budgerigar songs from samples (144). Zamansky et al.(145) provide an overview of ACI research methods, emphasizing the benefits to the ACI community of remaining open to methodologies from different fields.

### Literal Experience

**Figure 10:**
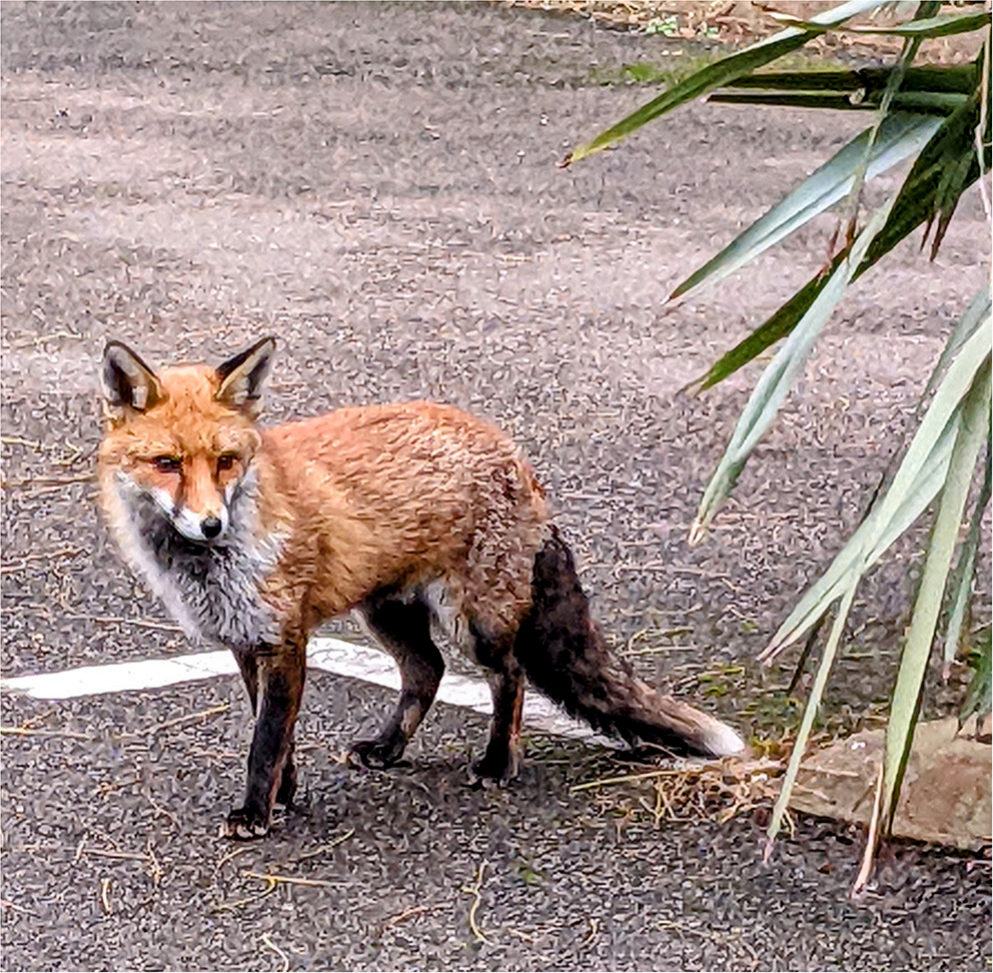
Urban fox in Battersea, UK.

A purely academic perspective can be quite limiting in regard to understanding the “other”, which is why some artists and researchers have deployed more imaginative techniques in their quests to understand the experiences of non-human animals and appreciate their aesthetic sensibilities. For example, there are adventurous researchers who have attempted to personally embody the life experiences of their non-human subjects in real time. One such explorer is Foster (87), who recounts his lived experiences of being a badger, an otter, a fox, a red deer and a swift in “Being a Beast” (see Figure 10: Urban fox). Foster has inhabited the same environments as the selected species and suggests that as he possesses similar sense receptors, he is able to draw parallels between his responses and theirs to a given situation. However, he also acknowledges that because all the signal processing is performed in the brain, phenomenological sensations might be different: “*The universe I occupy is a creature of my head. It is wholly unique to me*” (p. 8). Foster is interested in personal autonomy, identity and otherness, and has chosen to share his insights using an evocative writing style enriched with poetic language. His work is underpinned by extensive research; for example, into species-specific sensory modalities and somatotopic maps. Although there are fanciful passages where he postulates about the dreams of badgers and the non-chalance of otters, his work has an authority derived from him trying to live authentically as creatures in their natural habitat.

Thwaites also attempted to emulate a non-human species, by choosing to become a goat for a week (81). He proposed to explore the physicality of a goat's experience as part of a herd and his research led to the development of a goat exoskeleton so that he could experience life on the hoof. Thwaites commented: “*When I strapped on four legs, I couldn't use my hands, so my mouth became my interface with the world*” [in Pilcher, (146)]. He used technology to facilitate his performance as a goat, to the extent of wearing a device that could digest grass.

Foster's work was undertaken in a personal and private manner, then reflected upon and shared to allow others to vicariously experience his pleasures, trials and subsequent enlightenment. Thwaites' experiment was arguably an experimental performance art piece. Both were prepared to take risks in order to gain awareness of other species' sensibilities. They hoped that inhabiting the realm of the “other” would enable a deeper percipience of the possibilities and limitations associated with being a non-human animal; would help them to understand the animal's unique perspective; would allow them to assimilate the animal's natural environment in a corresponding way but through using their human senses. Yet after prolonged efforts, Foster claims he realized that he was incapable of creating a proper scent map because of his human dependency on vision. Both authors found their physical and perceptual limitations to be distracting during their intense engagement with their subjects' environment and lifestyle. In Art for Animals (147), which describes how contemporary artists have successfully included animals as participants and as audience members in their work, Fuller (148) highlights the problem faced by Foster and Thwaites, by asking: “*Is there a market for drugs that temporarily reconfigure nervous and perceptual systems to those of other species?*”

Fortunately, there are more practical and accessible methods for investigating non-human sensory experiences than Foster's and Thwaites' visceral adventures – for how many designers have a lifestyle that enables or motivates living in the woods for weeks eating worms or scaling a mountainside in prosthetics to chew cud?

### Close Relationships

Many humans have developed a close bond with a companion animal, and dog owners' combined insights have been used as a resource by Aspling et al. in their study “Understanding animals: a critical challenge in ACI” (137). In particular, Aspling et al. focus on owners pretending to be their dogs and posting on social media, which gives an indication of the kinds of thoughts that the humans imagine their dogs would share (about physical surroundings, weather, toys, treats, social lives and emotions).

“*it is not the taste of a leaf. that intrigues me. it is the crunch”* “*i heard there is a ball dropping later. does anybody have the details? i am interested in that”*- Thoughts of Dog, @dog_feelings, Twitter.

Helen MacDonald (149) painstakingly developed a relationship with a goshawk, Mabel. Although MacDonald never pretended to *be* a hawk, she describes the varying degrees of attachment and comprehension she felt as a result of her attentiveness to the “other” thus: “*I felt incomplete unless the hawk was sitting on my hand: we were parts of each other*.” Subsequently: “…*her world and my world are not the same, and some part of me is amazed that I ever thought they were*.” It is common for humans to feel strong affection for companion or tamed animals and vice versa (it seems); there are many narratives dealing with mutual understanding and apparently empathetic relationships.

### Narrative

For a population that is increasingly urban, increasing interest in reconnecting with non-human species is reflected in contemporary media. Big budget nature documentaries continue to be hugely popular, using narrative to engage the public with other lives. However, there has been scrutiny of the selective editing required to construct these stories. As filmmaker Simon Cade says*: “…they just choose a few moments that provide the maximum emotional impact”* (150). A different style of documentary can be seen in “*Stray*” (151), filmed in Instanbul and shown entirely through the perspective of its street dogs. The creators state that the film “*explores what it means to live as a being without status or security*”. Although this explicitly references the dogs themselves, the film also implicitly portrays human society, offering an example of animals being used as ciphers to explore human psychology.

Perhaps Aesop's Fables, a collection of folktales from Ancient Greece, is the earliest well-known example of anthropomorphism by storytellers. The behaviors of the animal protagonists are metaphors for human behaviors and the narratives are designed to express moral values. This tradition continues to the present day in children's literature, where one of the strengths of anthropomorphism is that it avoids the problem of human representation and therefore makes the text universally relevant. Fantasy fiction for older audiences also draws on folklore and mythology; popular modern examples include Pullman's “*His Dark Materials”* (152) and Martin's “*Song of Ice and Fire*” (153). Pullman envisages a world of people imbued with dæmons, who are human souls embodied as animals, similar in concept to spirit animals; skinchangers (humans who can enter the mind of another animal) are fundamental to Martin's plot. These human-animal connections reference the ancient tradition of shamanism that connects people with nature through interaction with spirits and is believed to have originated with hunting and gathering communities. A person's spiritual journey in this context is often facilitated by a spirit animal guide, but although the attributes of the animal influence their perceived guidance (e.g., a bear is emblematic of strength, an eagle epitomizes vision), the animals seem to be used symbolically.

Science fiction offers writers scope to experiment with different frames of reference. As a case in point, Tchaikovsky, in “*Children of Time”*, writes from the perspective of an evolved spider, here seeing a human spaceship for the first time: “*Every detail is bizarre and disturbing, an aesthetic arising from the dreams of another phylum, a technology of hard metal and elemental forces*” (154). Tchaikovsky excels in evoking the spider's alien consciousness; she conceptualizes the world in spiraling networks of interconnectivity with her sisters, speaks with vibrations and is able to discuss maths with other species such as stomatopods and humans. Despite, or perhaps due to, being a fictional account, the work successfully introduces human readers to novel sensibilities. Nonetheless, Westerlaken, in Imagining Multispecies Worlds, brings home the importance of actually sharing a world space with other species for gaining empathy: “*Stories will always lack some of the sensorial engagement of the experiences themselves*” (155).

In contrast to a traditional linear narrative approach, completely new dimensions of experience are being explored through the use of immersive technology.

### Immersion

In the world of games, “*Pigeon Simulator*” from TinyBuild (https://www.pigeonsimulator.com/) is described as a “physics sandbox roguelike” where players embody (antagonistic) city pigeons. Blue Twelve Media (https://stray.game/) are releasing an adventure game (also) called “*Stray*” in 2022, where the player is represented as a cat who interacts with the world from a feline perspective.

In human scenarios, there have been attempts to use VR (virtual reality) technology to enable people in caring roles to empathize more strongly with their patients. For example, VR has been used as a tool to empower nurses and family members, allowing them to experience the world as those in their care might experience it (156–158). However, Martingano et al. (159) discovered that VR seems to improve emotional, but not cognitive empathy, meaning that it can arouse compassion, but fails to help users understand the perspective of another. They suggest that cognitive empathy requires “*…more effortful engagement, such as using one's own imagination to construct others' experiences*.” McFarland's view in (160) was: “*No film-maker, or virtual reality expert, could convey to us what it is like to be a bat, no matter how much they knew about bats*.” While this view is apposite, McFarland acknowledged that although qualia are subjective qualities, if humans have experienced the same sensations as each other, they usually have a common understanding, despite each person being unique in their internal processing of the information (160).

Extending VR applications to support humans in their understanding of animals has already had some success. Recent ACI work in this area includes the creation and deployment of VR videos that express the visual experiences of (i) turtles and tortoises, (ii) cats and dogs and (iii) frogs and geckos (161). The focus is on showcasing alternative color spectra and dynamic vision, and the research motivation is to provide opportunities for humans to learn about animal vision in order to gain appropriate design perspectives. As humans are typically so dependent on vision, highlighting differences in visual perception between species may be a critical aspect of understanding the other.

Hook (162) developed a wearable horse-shaped head with lenses that enabled humans (who have bifocal vision) to view their surroundings as if their eyes were situated on either side of their head. This provided a typical prey species perspective, providing a much larger field of view. North (163) also explored the use of horse adaptations (robotic ears) worn by humans in order to further their understanding of horse communication using ear movement signals. Even though the ear movements are perceived by conspecifics as visual signals, North's work highlights the fact that interaction modalities vary from species to species. Hook describes his method for this project as *speculative design*, which emphasizes critical reflection around the future implications of a design, often using design fictions to provoke discussion (164). North, meanwhile, refers to his work as *science fiction autoethnography*.

Both these example projects by Hook and North required *expert crafting* in order to recreate the perception and anatomical features that are used by the animal, so that humans might gain deeper understanding of a horse's experience.

### Craft

Crafting has a visceral, multisensory quality. It is related to fabrication or making, but with a stronger emphasis on exploring the materiality of the crafted object and the confluence of modalities that give rise to our perception of it. Craft has the potential to enhance the designer's sensory and intellectual appreciation of form and substance, which are attributes of an object that may have aesthetic appeal. In design work with elephants, French et al. (142) adopted a Research though Design *and Craft* methodology, where the crafting aspect was a fundamental aspect of negotiating an interactive enrichment design that would be appropriate for an elephant – not only designed according to an elephant's cognitive and physical abilities, but one that would be both pleasurable and engaging. The project started with ideas borrowed from game design and knowledge of an elephant's sensory modalities, then crucially, the researchers discovered that craft offered a physical way to mediate between designer and user through *mutual interactions with the same object*.

“*Craft is the outputs from my brain through material practice by using my hands – the opposite to inputs such as reading, watching, listening … When we output something physically, we learn so much through all our senses.”* – Mori (165) artist and metalworker (from Craft Council Stories, 2020).

Craft connects the designer with the aesthetic properties of the crafted object by promoting both cognitive and multi-sensory appreciation. Handling an object gives rise to insights regarding its aesthetic dimensions. Similarly, *tinkering* with electronics (included within the practice of craft) is more fruitful for developing an appreciation of the sensors and actuators used in interactive systems than using off-the-shelf solutions. Synthesized outputs are not objects – yet they can be concrete, perceivable experiences, such as sounds and vibrations. Therefore, they have aesthetic dimensions that are both discernable and potentially controllable by both humans and non-humans. The profound experiential knowledge gained from physical interaction with an object is something shared between designer and user, despite their reliance on different modes of perception.

These examples of creative methods used by humans to extend their aesthetic sensibilities and embrace the experiences of other animals hopefully serve to show how artistic perspectives can be inspirational for and complementary to scientific investigations in this field. Baker, in the introduction to Artist Animal, comments: “…*art has the potential to offer a distinct way of framing or unframing issues*…” (166). In this context, perhaps it is the *unframing* that is crucial, facilitating our ability to imagine a different way of knowing the world.

## Conclusions

“*We are human only in contact, and conviviality, with what is not human.”* – David Abram (7) (reprint 2017).

Humans are gaining an improved ecological perspective on the environment and their co-inhabitants using a combination of science, technology and imagination. We observe and interpret, use tools to derive more knowledge, and create fictional or metaphorical narratives that attempt to explain our existence.

One aspect of the human quest to understand everything is our desire to understand other animals. Human society facilitates communication and shared intelligence between human individuals but gaining awareness of what it is like to be another species is more challenging and controversial, requiring a combination of scientific investigation, insight and imagination. Current studies indicate that aesthetics are fundamental aspects of the experiences of all living creatures and should therefore be taken into consideration by the designers of those experiences, as well as designers whose work occupies a multi-species-shared environment. A deeper awareness of the aesthetic experiences of non-humans can support human design endeavors by increasing sensibility to the environmental and ecological effects of human activities.

This paper has attempted to address ideas about different dimensions of being, by exploring and expanding notions of aesthetic sensibility. In *2. Rationale for aesthetics*, reasons for the existence of aesthetic sensibility were discussed from different disciplinary perspectives. *3. Perception, aesthetic sensibility and behavior* offered a review of current work on animal perception, pointing to sensory modalities that are important for designers to consider. This section also suggested some intensely rewarding behaviors exhibited by different species that may be good candidates for holistic aesthetic appreciation – being more than the sum of the individual senses involved. Finally, *4. Interaction Design: exploring aesthetics* comprised a collection of ways in which humans have engaged creatively with the sensory and cognitive experiences of other species. This was presented as a set of suggestions to support interaction designers to better understand their non-human users (intended or otherwise) and to design with confidence and respect.

Remaining open-minded and receptive to non-human perspectives and abilities has the potential to enhance human lives, by opening the doors to novel and mysterious aesthetic experiences. Through an exploration of difference, not only do we gain more insight into other species, but we may also learn more about the aesthetic sensibilities that we have in common. And indeed, by embracing alternative ways of being, we are extending inclusivity beyond human culture and personal identity.

## Author Contributions

The author confirms being the sole contributor of this work and has approved it for publication.

## Conflict of Interest

The author declares that the research was conducted in the absence of any commercial or financial relationships that could be construed as a potential conflict of interest. The handling editor declared a past supervisory role and a past co-authorship with the author, FF.

## Publisher's Note

All claims expressed in this article are solely those of the authors and do not necessarily represent those of their affiliated organizations, or those of the publisher, the editors and the reviewers. Any product that may be evaluated in this article, or claim that may be made by its manufacturer, is not guaranteed or endorsed by the publisher.
